# Six years of experience in entomological surveillance of indoor residual spraying against malaria transmission in Benin: lessons learned, challenges and outlooks

**DOI:** 10.1186/s12936-015-0757-5

**Published:** 2015-06-12

**Authors:** Martin C. Akogbéto, Rock Y. Aïkpon, Roseric Azondékon, Gil G. Padonou, Razaki A. Ossè, Fiacre R. Agossa, Raymond Beach, Michel Sèzonlin

**Affiliations:** Centre de Recherche Entomologique de Cotonou (CREC), 06 BP 2604 Cotonou, Bénin; Faculté des Sciences et Techniques, Université d’Abomey Calavi, Cotonou, Bénin; Université du Massachusetts Amherst, Amherst, MA USA; Université d’Agriculture de Kétou, Kétou, Benin; Center of Disease Control, Atlanta, USA

**Keywords:** Indoor residual spraying, Lessons learned, Benin

## Abstract

**Background:**

From 2008 to 2013, a prevention intervention against malaria based on indoor residual spraying (IRS) was implemented in Benin. From 2008 to 2012, Ficam M®, a bendiocarb-containing product was used for house spraying, in association with pirimiphos methyl EC (Actellic EC) in 2013. This operation aimed to strengthen the effectiveness of treated nets so as to expedite the achievement of Millennium Development Goals (MDGs): the reduction of morbidity and mortality due to malaria by 75 % from 2000 to 2015.

**Methods:**

Monitoring and evaluation (M&E) was implemented in order to evaluate the impact of IRS intervention on malaria transmission. *Anopheles gambiae s.l*. populations were sampled by human landing catch. In addition, window exit traps and pyrethrum spray catches were performed to assess exophagic behaviour of *Anopheles* vectors the main malaria vector in the treated areas. The residual activity of insecticide in the treated walls was also assessed using WHO bioassay test.

**Results:**

The purpose of this project was to draw attention to new challenges and future prospects for the success of IRS in Benin. The main strength of the intervention was a large-scale operation in which more than 80 % of the houses were treated due to the strong adhesion of population. In addition, a significant reduction of the EIR in areas under IRS were observed. However, there were many challenges including the high cost of IRS implementation and the identification of suitable areas to implement IRS. This was because of the low and short residual effect of the insecticides recommended for IRS and the management strategy for vector resistance to insecticides. This indicated that challenges are accompanied by suggested solutions. For the cost of IRS to be accessible to states, then local organizations need to be created in partnership with the National Malaria Control Programme (NMCP) in order to ensure relevant planning and implementation of IRS.

**Conclusion:**

As an anticipatory measure against vector resistance, this paper proposes various methods, such as periodic IRS based on a combination of two or three insecticides of different classes used in rotation every two or three years.

## Background

In 2008, Benin committed, for the first time, to implementing an IRS campaign in order to protect its population against malaria. Such a strategy was chosen to strengthen the action of the existing long-lasting insecticidal Nets (LLINs) as first prevention strategy against malaria. Indeed, Benin had signed up to the Millennium Development Goals (MDGs), which components included a 75 % reduction of malaria morbidity and mortality between 2000 and 2015. To achieve this level of malaria reduction within 15 years, an integrated control based on the simultaneous use of multiple strategies against the vector and the parasite was necessary. In Africa, particularly in Benin, the first mass preventative method effectively applicable against mosquito bites is the use of LLINs [1, 2]. A great deal of progress has been made in Benin as far as the use of this preventative method is concerned. The rate of LLIN use by pregnant women and children under five increased from 32 % in 2006 to 60 % in 2010 and 70 % in 2013 through a mass distribution [3, 4]. Similar progress has been noted regarding the implementation of indoor residual spraying (IRS). The proportion of the African population at risk of malaria but protected by IRS increased from 5 % in 2005 to 11 % in 2011 [5]. As documented in recent World Malaria Reports, reductions in malaria disease burden have coincided with the massive scale-up of malaria prevention measures, particularly the use of LLINs and IRS [5, 6]. The National Malaria Control Programme (NMCP) of Benin has noted that IRS is a promising intervention in reducing the malaria burden. Some African countries, particularly Zimbabwe, Namibia, Swaziland, Botswana, and Mozambique have had a reduction in malaria transmission and parasite rates of *Plasmodium* in children [7] with prolonged IRS campaigns over several decades. Maxwell *et al*. [8] also observed a decrease in anaemia and child mortality rates resulting from the massive use of impregnated nets (ITNs). According to Lengeler [1], 5.5/1,000 children are saved due to sleeping under ITNs.

Malaria elimination is conceivable; it will require a lot of political will, effort and expertise.

The IRS campaign started in Benin in 2008 with the support of The President’s Malaria Initiative (PMI). From 2008 to 2010, over 315,000 people out of 350,000 were protected per year by the intervention in Ouémé Department, south of Benin. From 2011 to 2013, the operation was moved from Ouémé to Atacora (northern Benin) where over 650,000 people per year were protected. Due to vector resistance to pyrethroids across Benin, two insecticides of two different classes were used in rotation: bendiocarb (a carbamate) and pirimiphos methyl EC (an organophosphate).

NMCPs in Africa usually implement good control strategies; however, despite impressive reductions in malaria transmission mainly through LLIN, IRS and access to treatment it is recognized that, evaluation and routine vector surveillance is a weak component of many national disease control programmes. Vector surveillance should be increased prominence in the planning, implementation, monitoring, and evaluation of vector control interventions. The goal of this study was to evaluate entomological impact following large-scale protection through the spraying in two departments of Benin. This evaluation focused on the decay of insecticide on treated walls, the population dynamics of *Anopheles gambiae,* the level of entomological inoculation rate (EIR), and the development of vector resistance in areas under intervention compared to control areas. This document is not a collection of impact indicators, it also leveraged six years of IRS tracking experience in the field to identify the new challenges. Suggestions are maked to address them and views on the future of IRS in Benin are given.

## Methods

### Intervention areas

**Fig. 1:**
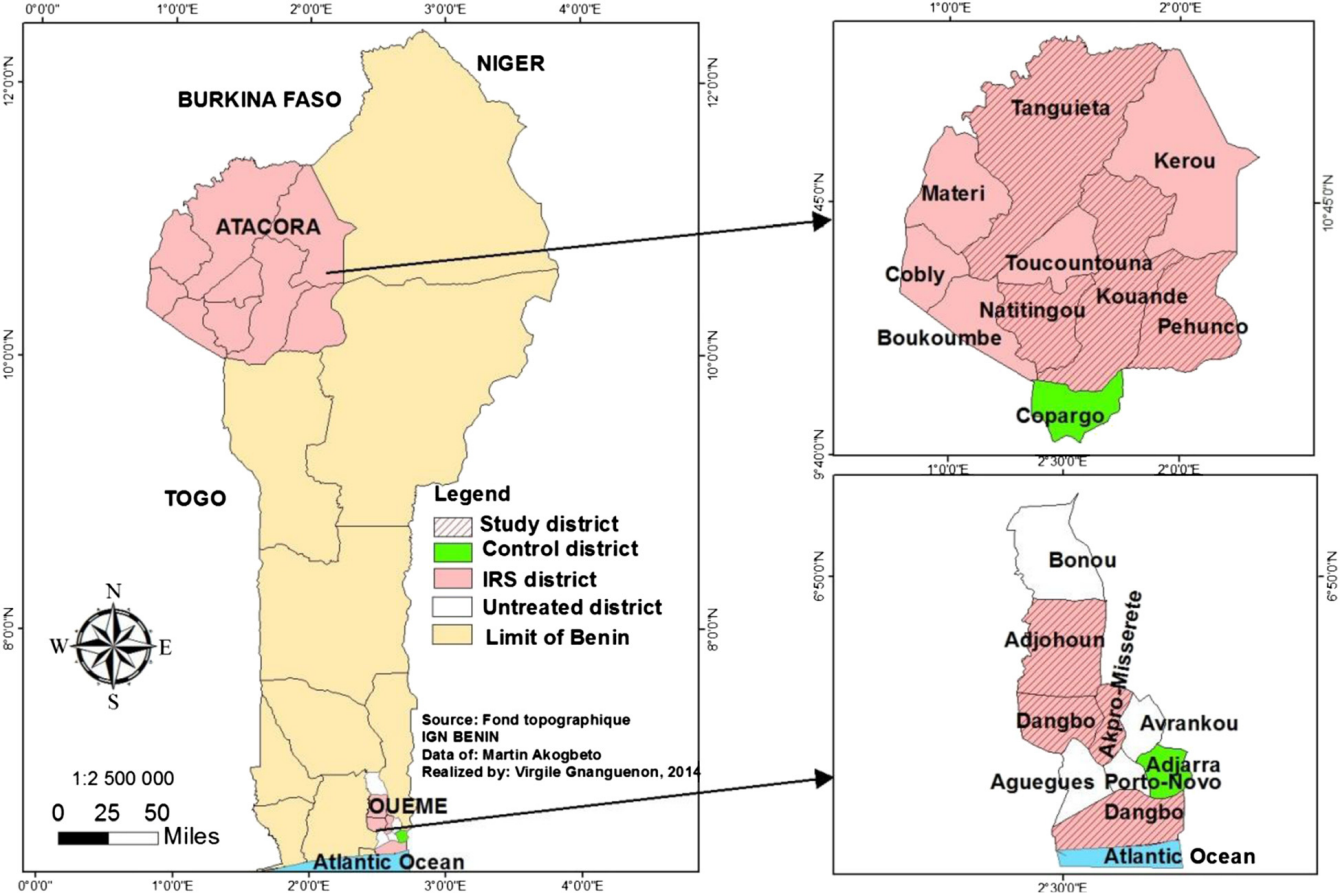
Map of Benin showing the districts under IRS and those under Monitoring-Evaluation (study districts)

Two IRS campaigns were implemented by the NMCP from 2008 to 2013. In 2008, IRS was conducted in Ouémé Department in southeast Benin (Fig. 1), protecting 350,000 people. Four out of the nine districts in that department were involved in this operation: Adjohoun, Dangbo, Sèmè, and Missérété.

South of Benin is characterized by two dry seasons (December to March and August to September) and two rainy seasons (April to July and October to November). The existence of two rainy seasons in the year results in an almost year-round presence of *Anopheles* and permanent malaria transmission. Given the short, residual action of insecticides currently available for IRS, the spraying has to be conducted at least twice a year in order to expect a significant decrease in *Anopheles* density throughout the year. Unfortunately, the cost of IRS operation makes it impossible for Benin to afford spraying twice a year.

Based on the year-round transmission and presence of malaria and its vector due to the two rainy seasons per year, IRS was discontinued in Ouémé and moved to Atacora Department, north of Benin in 2011. This department is characterized by one dry season (November to May) and one rainy season (June to October). Some 650,000 inhabitants were taken into account in the nine districts (Natitingou, Tanguieta, Toucountouna, Materi, Boucombe, Cobly, Kouandé, Péhunco, and Kerou) that were covered by the operation. In Atacora, the major economic activity is agriculture, mainly cotton and millet, for which various classes of pesticides (pyrethroid, carbamate, organophosphate) are used. In contrast, the main activities in Ouémé include trade, corn agriculture and fishing facilitated by the presence of Ouémé River. The M form of *An. gambiae,* recently named *Anopheles coluzzii* [9], is the major malaria vector in Ouémé and the S form named *An. gambiae* [9] in Atacora.

### Control areas

In the Department of Ouémé, Adjara, a peripheral area of Porto-Novo (administrative capital of Benin), was selected as a control (Fig. 1). Since all of the Atacora districts were to be involved in the campaign, the control district (Copargo) was selected in Donga, a department close to Atacora. The distance between Copargo and the nearest IRS district in Atacora (Kouandé) is 15 km (Fig. 1). The population was not denied net use either in intervention districts or control districts.

### Intervention implementation

Due to the multiple resistance of *An. gambiae* to insecticides described in Benin [10–14], IRS campaigns were preceded by two studies in order to select the best insecticides.

The first study was an evaluation of various insecticides in experimental huts in which three: Sumithion® 40 WP (Fénitrothion, an organophosphate), Master Quick ZC (mixture of chlorpyriphos 250 g/l + deltamethrin 12 g/l), and Ficam M® (bendiocarb 800 g/kg, a carbamate), proved to be the most effective against pyrethroid-resistant *An. gambiae* [15]. However, after this evaluation, bendiocarb was the only product selected by the NMCP since Master Quick ZC formulation did not have World Health Organization Pesticide Evaluation Scheme (WHOPES) recommendation to be used in public health programmes. Sumithion® 40 WP was excluded due to doubts about its safety in terms of secondary effects as well as its odour [16].

After two years of bendiocarb use in the north, a resistance of *An. gambiae* to this product was noted. Then, an experimental hut trial comparing the effectiveness of the three classes of insecticides (one carbamate (bendiocarb), two organophosphates (fenitrothion and pirimiphos methyl) and one pyrethroid (lambdacyalothrin) was conducted against wild, free-entered, resistant *An. gambiae s.l.* population to pyrethroids. After this trial, the NMCP selected pirimiphos methyl as alternative insecticide to be used in rotation with or as an alternative to bendiocarb.

The two IRS campaigns (Ouémé and Atacora) were supported by the PMI, funded by the US Government. The technical implementation was conducted by two technical partners: Research Triangle International (RTI) from 2008 to 2011 and Abt Associates from 2012 to 2014. The M&E on the effectiveness of the intervention was carried out by *Centre de Recherche Entomologique de Cotonou* (CREC). Over 80 % of households were treated in the two regions (Oueme and Atacora) [16, 17]. In Oueme,, a bendiocarb-containing product was used, and in Atacora, Ficam M® and Actellic 50 EC, a pirimiphos methyl-containing product were used in rotation.

### Mosquito sampling, laboratory processing and parameters studied

In each district under M&E, two villages were selected, and two houses chosen per village for mosquito collection to monitor malaria transmission. Monthly, mosquito collections were carried out by human landing cathe from 9 pm to 5 am inside and outside houses using a mouth aspirator, by resident volunteers who had previously given consent. Every month, two nights of mosquito collections were carried out. All female mosquitoes belonging to *An. gambiae* complex were identified based on morphological characteristics using standard identification keys [18]. Vector species were dissected using a microscope to determine parous rates. Head-thoraxes were tested using enzyme-linked immunosorbent analysis (ELISA) according to Wirtz *et al.* [19] for the presence of circumsporozoite protein (CSP) of *Plasmodium falciparum*, the major malaria parasite occurring in the study area. The recorded data were used to assess the feeding behaviour (HBR) of mosquitoes, the physiological age and the EIR of the vectors.

In order to assess the impact of the interventions on blood feeding inhibition of mosquitoes in treated houses, sampled mosquitoes were sampled using window exit traps and morning pyrethrum spray catches (PSC). These two sampling methods led to an accurate estimation of the total density of mosquito species in the treated houses and the proportion of female mosquitoes exiting from the houses. *Anopheles* mosquitoes collected were classified according to the state of their abdomens.

### Insecticide susceptibility tests

Larvae of *An. gambiae* collected in Atacora were reared until they were two to five days old. These mosquitoes were assayed using World Health Organization (WHO)-determined dosage of papers impregnated with bendiocarb 0.1 %. Four batches of 25 unfed females were exposed for 60 min at 27 ± 1 °C and 80 % relative humidity. Twenty-five *An. gambiae* females were introduced into each tube and monitored at different time intervals (10, 15, 20, 30, 45, 60 min); the number of ‘knocked-down’ mosquitoes was recorded. After one hour of exposure, mosquitoes were transferred into holding tubes with cotton wool saturated with a 10 % honey solution. Batches of mosquitoes exposed to untreated papers were used as control. Mortalities were recorded after 24 h and the susceptibility status of the population was graded according to WHO protocol [20]. Dead and surviving mosquitoes from this bioassay were then kept separately in Eppendorf tubes containing silica gel and finally stored at –20 °C for further molecular analysis by PCR.

### Data analysis

The resistance status of mosquito samples was evaluated according to the WHO criteria [20]:

(i) Mortality rate is >98 %: the population is considered fully susceptible,
(ii) Mortality rates 90–98 %: resistance is suspected in the population,
(iii) Mortality rates <90 %, the population is considered resistant.

To compare the status of bendiocarb resistance, Fisher’s exact test was carried out to determine if there was any significant difference between mortality rates of populations of *An. gambiae s.s.* for 2010 to 2012. Furthermore, the human biting rate [number of bites/man/night] (ma), the sporozoite rate (Is) and the entomological inoculation rate (EIR) were determined. The reduction of EIR in the treated districts compared to the control were evaluated. Blood feeding rates were calculated. Comparisons of these rates were made by the Chi-square test.

### Ethical consideration

Ethical approval for this study was granted by the Ethical Committee of the Ministry of Health in Benin. The mosquito collectors gave prior informed consent and they were vaccinated against yellow fever. They were also subjected to regular medical check-ups with preventive treatments of malaria.

## Results

### Decrease in malaria transmission in Ouémé Department

#### Results after one round of spraying (2009)

In April 2009, IRS took place in the department of Ouémé at the beginning of the rainy season. The operation aimed to protect the population of Adjohoun, Dangbo, Missérété, and Sèmè against mosquito bites during the long rainy season (April to July). As expected, mosquito bite frequency decreased significantly in the IRS area during this period measured by human landing catch. Taking into account the total number of *An. gambiae* collected from April to July in the four districts under IRS, an individual was barely bitten more than 2.2 times per night (708/320). This biting rate was very low in Adjohoun, Dangbo and Missérété where it was 1, 0.25 and 0.9, respectively. However, in Adjarra (control), *Anopheles* bite frequency was significantly higher (14 bites per person per night). The relatively high rate observed in Sèmè (7.92) despite house treatment with bendiocarb, was due to the environment of this district. It is appropriate for mosquito breeding due to the presence of numerous swamps, clay soil that holds water after any light rain and a very shallow water table. One of the consequences of the reduction in man-vector contact in districts under insecticide treatment (reduction of over 90 % in Adjohoun and Dangbo and 43.42 % in Sèmè, Table 1) was the significant decline in *Anopheles* carrying *P. falciparum*. For four months of catches, no *Anopheles* was found positive for CSP antigen of *P. falciparum* at Adjohoun, Dangbo or Sèmè after examining 380 *An. gambiae* using ELISA. Nevertheless, in the control area, the sporozoite rate was 6.34 % (Table 1).

**Table 1:** Human biting rate (HBR) and entomological inoculation rate (EIR) observed in *Anopheles gambiae* during the IRS coverage period (rainy season) in the treated and control areas, Oueme, 2009

	Adjohoun	Dangbo	Missérété 1	Missérété 2	Sèmè	Total (treated)	Adjara (control)
Total caught	64	16	0	121	507	708	896
Man night	64	64	64	64	64	320	64
HBR (b/m/n)	1	0,25	0	1.89	7.92	2.21	14
Total tested (ELISA)	64	16	0	121	300	501	896
S (%)	0	0	0	0.84	0	0.2	6.34
EIR (pi/h/n)	0	0	0	0.02	0	1	0.89
Réduction (HBR) (%)	92.86	98.21	-	86.50	43.42	84.21	-
Réduction (EIR) (%)	100	100	-	98.21	100	96.84	-

From the analysis of these data, it appears that the transmission expressed in terms of EIR was close to zero in the first four months after IRS. In the same period, it was estimated that each Akron inhabitant (control area) received, overall, 0.89 infected bites per night (26.7 *P. falciparum*-infected bites per month). These results show that for four months treating houses with bendiocarb had been very effective. Data analysis did not go beyond four months because the fifth and sixth months (August and September) were part of the dry season when mosquito density is low in the majority of districts. In addition, several studies have shown that, in general, the effectiveness of bendiocarb hardly exceeds three to four months [15, 21–25].

#### Results after two successive rounds of IRS (2010)

**Fig. 2:**
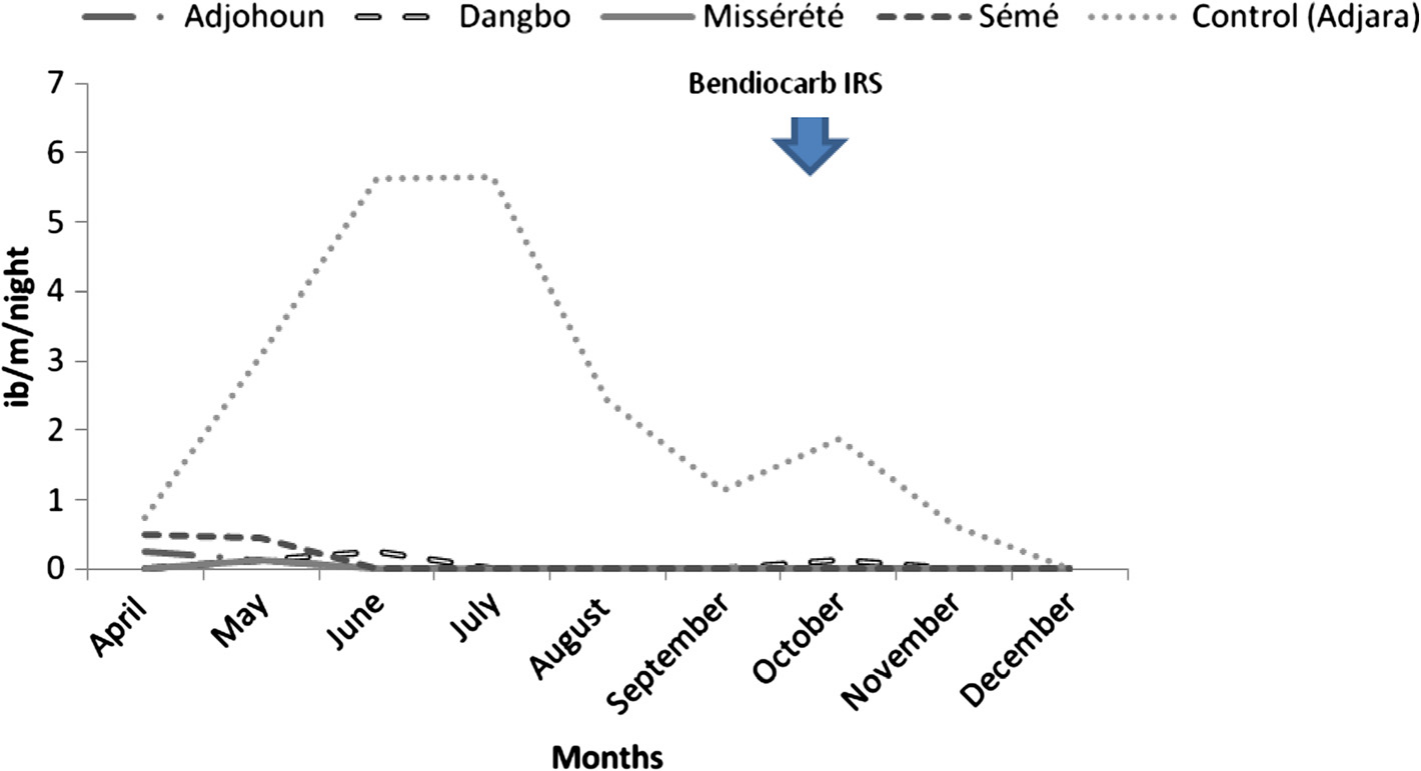
Monthly variation of Entomological Inoculation Rate after IRS implementation in 4 districts and in the control area in 2010, department of Ouémé, Benin

In 2010, two IRS rounds were completed. The two rounds were implemented in April (beginning of the long rainy season) and then in August (prior to the beginning of the short rainy season) (Fig. 2). Figure 2 shows that in the control area (Akron), there were two malaria transmission peaks, one between June (EIR = 5.63 infected bites/man/night) and July (EIR = 5.64 bites/man/night), the other in October (EIR = 1.88). A projection of this data throughout 2010 shows an extremely high EIR in the control area (821.25 infected bites/man/year (2.25 × 365). In addition, transmission remained barely noticeable throughout the year in the four districts under treatment (Fig. 2 and Table 2). Annual EIRs in these areas were significantly lower than those in the control area: 821.25 in the control against 10.95 (0.03 × 365) (Table 2) in Adjohoun (a 98.7 % reduction), 7.3 in Dangbo (99 % reduction) and 55.1 in Sèmè (93 % reduction). The advantage of two IRS rounds a year is to keep steady pressure control on the density of *Anopheles*. However, the NMCP could not continue the IRS campaign at this rate, considering the cost and burden of the strategy. It was therefore decided in 2011 to move the IRS to Atacora, a department characterized by a single rainy season each year.

**Table 2:** Human biting rate (HBR) and entomological inoculation rate (EIR) observed in *Anopheles gambiae* during the indoor residual spraying period in the treated and control areas, Oueme, 2010

Locations	Parameters	April (IRS)	May	June	July	August (IRS)	September	October	November	December	Total	EIR (ib/m/year)
	n	5	3	4	7	2	0	5	7	0	33	12.91
	man night	4	8	8	8	4	4	8	8	4	56	
Adjohoun	HBR (b/m/night)	1.25	0.38	0.50	0.88	0.50	0	0.63	0.88	0	0.59	
	IS	0.20	0.33	0	0	0	-	0	0	-	0.06	
	EIR (ib/m/night)	0.25	0.13	0	0	0	-	0	0	-	0.04	
	n	2	19	77	8	0	1	11	3	1	122	23.86
	man night	4	8	8	8	4	4	8	8	4	56	
Dangbo	HBR	0.50	2.38	9.63	1.00	0	0.25	1.38	0.38	0.25	2.18	
	IS	0	0.05	0.03	0	-	0	0.09	0	0	0.03	
	EIR/night	0	0.13	0.25	0	-	0	0.13	0	0	0.07	
	n	6	11	24	15	2	3	17	14	1	93	6.06
	man night	4	8	8	8	4	4	8	8	4	56	
Missérété	HBR	1.50	1.38	3.00	1.88	0.50	0.75	2.13	1.75	0.25	1.66	
	Is	0	0.09	0	0	0	0	0	0	0	0.01	
	EIR/night	0	0.13	0	0	0	0	0	0	0	0.02	
	n	21	163	262	278	29	6	71	16	0	846	55.14
	man night	4	8	8	8	4	4	8	8	4	56	
Sémé	HBR	5.25	20.38	32.75	34.75	7.25	1.50	8.88	2.00	0	15.11	
	IS	0.10	0.02	0	0	0	0	0	0	-	0.01	
	EIR/night	0.50	0.46	0	0	0	0	0	0	-	0.15	
	n	35	212	473	397	215	51	132	58	7	1580	926.84
	man night	4	8	8	8	4	4	8	8	4	56	
Control (Adjara)	HBR	8.75	26.50	59.13	49.63	53.75	12.75	16.50	7.25	1.75	28.21	
	IS	0.09	0.12	0.10	0.11	0.05	0.09	0.11	0.09	0	0.09	
	EIR/night	0.75	3.08	5.63	5.64	2.44	1.13	1.88	0.63	0	2.54	

IS: sporozoitic index

#### Duration of Bendiocarb and pirimiphos methyl EC action on houses in Atacora (years 2011–2013)

**Fig. 3:**
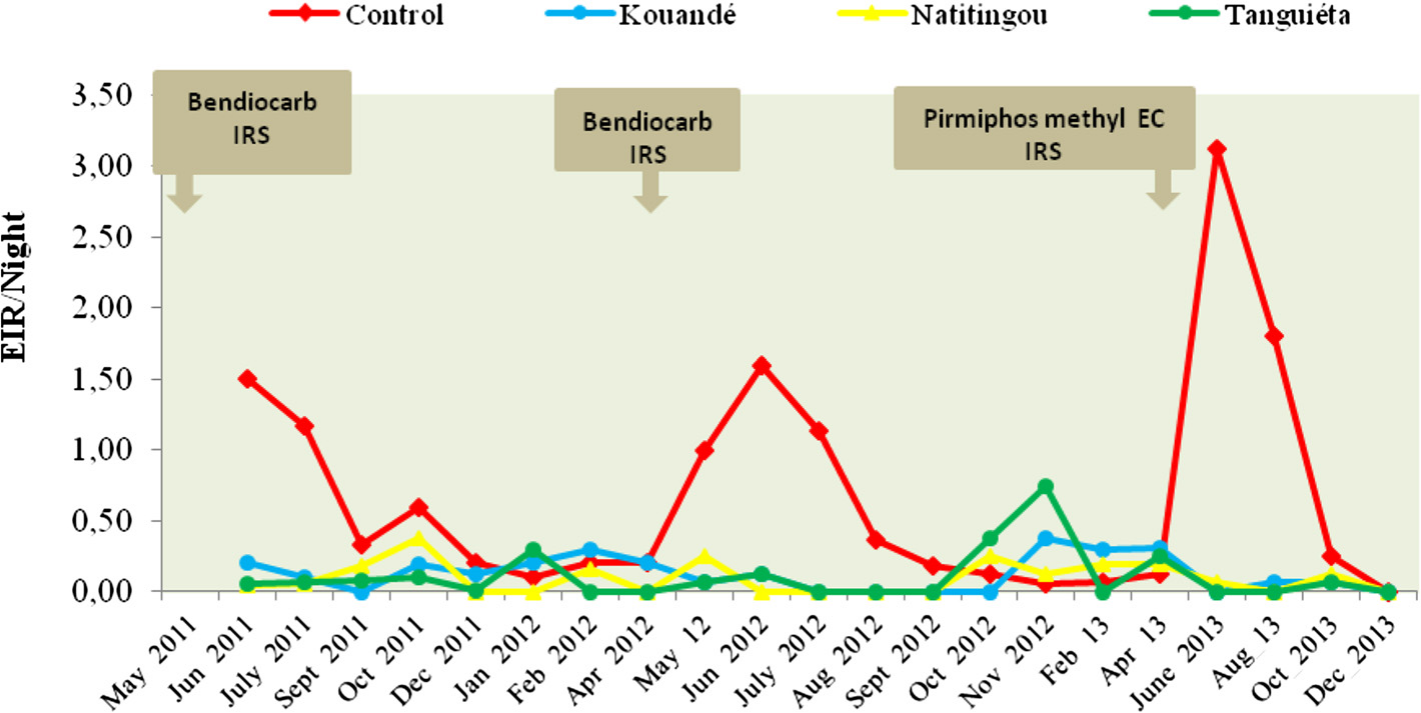
Monthly variation of EIR from 2011 to 2013 after each round of IRS in districts under IRS (Kouandé, Natitingou, Tanguiéta) *versus* the control district

Figure 3 and Tables 3, 4 and 5 shows the change in the EIR from 2011 to 2013, a period during which three IRS rounds were made for three years. Each IRS round was carried out in May or April prior to the rainy season. In 2011 and 2012, the houses were treated with Ficam M® (bendiocarb 800 g/kg, a carbamate) and in 2013 with Actellic 50 EC (pirimiphos methyl, an organophosphorus). The data in Fig. 3 are related to three districts (Natintingou, Tanguieta and Kouandé) under IRS and the control area (Copargo). Overall, the transmission curve has the same shape from one year to the next: a significant decrease in the EIR for four months (May to September) after spraying, then an increase from the fifth month. This applies to both insecticides in the three-year period. It also shows that efficiency of bendiocarb and pirimiphos methyl CS hardly exceeds four months.

**Table 3:** Variation of entomological inoculation rate (EIR) in *Anopheles gambiae* in districts under indoor residual spraying (Kouandé, Natitingou and Tanguiéta) *versus* the control (Copargo) in 2011

Districts	Parameters	July_2011	Sept_2011	Oct_2011	Dec_2011
Copargo (Control)	IS	0.09	0.06	0.68	3.33
	HBR/night	12.94	5.25	0.88	0.06
	EIR/night	1.16	0.33	0.60	0.20
	EIR/month	34.93	9.95	18.00	6
Tanguiéta	IS	0.33	0.04	0.19	0.17
	HBR/night	0.19	2.19	0.53	0.06
	EIR/night	0.06	0.08	0.10	0.01
	EIR/month	1.86	2.43	3	0.30
Natitingou	IS	0.17	0.15	0.13	0
	HBR/night	0.31	1.19	3.00	0
	EIR/night	0.05	0.18	0.38	0
	EIR/month	1.59	5.36	11.25	0
Kouandé	IS	0.12	0.00	0.23	0.17
	HBR/night	0.83	2.18	0.81	0.75
	EIR/night	0.10	0	0.19	0.13
	EIR/month	3	0	5.61	3.75

**Table 4:** Variation of entomological inoculation rate (EIR) in *Anopheles gambiae* in districts under indoor residual spraying (Kouandé, Natitingou and Tanguiéta) *versus* the control (Copargo) in 2012

Districts	Parameters	Jan_12	Feb_12	Apr_12	May_12	Jun_12	July_12	Aug_12	Sept_12	Oct_12	Nov_12
Copargo	IS	1.67	1.05	1.60	0.11	3.20	0.04	0.57	0.03	0.24	1.00
	HBR/night	0.06	0.19	0.13	8.94	0.50	3.00	0.63	5.94	0.50	0.06
	EIR/night	0.10	0.20	0.20	1.00	1.60	0.13	0.36	0.18	0.12	0.06
	EIR/month	3	6.00	6.00	30	48.00	3.75	10.80	5.40	3.60	1.80
Tanguiéta	IS	5	0	0	0.07	0.15	0	0	0	0.21	0.27
	HBR/night	0.06	0.06	0	0.94	0.81	0.50	0.63	5.38	1.75	2.75
	EIR/night	0.30	0	0	0.06	0.12	0	0	0	0.38	0.75
	EIR/month	9.00	0	0	1.88	3.74	0	0	0	11.25	22.50
Natitingou	IS	0	0.11	0	0.06	0	0	0	0	0.07	0.17
	HBR/night	0	1.50	0	4.50	0.56	0.63	1.06	3.44	3.44	0.75
	EIR/night	0	0.16	0	0.25	0	0	0	0	0.25	0.13
	EIR/month	0	4.80	0	7.50	0	0	0	0	7.51	3.75
Kouandé	IS	3.33	5.00	1.60	0.02	0.05	0	0	0	0.00	0.20
	HBR/night	0.06	0.06	0.13	3.56	2.63	0.44	1.13	4.56	0.56	1.88
	EIR/night	0.20	0.30	0.20	0.06	0.13	0	0	0	0	0.38
	EIR/month	6	9.00	6.00	1.87	3.76	0	0	0	0	11.28

**Table 5:** Variation of entomological inoculation rate (EIR) in *Anopheles gambiae* in districts under indoor residual spraying (Kouandé, Natitingou and Tanguiéta) *versus* the control (Copargo) in 2013

Districts	Parameters	Feb_13	Apr_13	Jun_13	Aug_13	Oct_13	Dec_13
(Copargo) Control	IS	0.25	0.20	0.20	0.52	0.08	0
	HBR/night	0.25	0.63	15.69	3.44	3.25	0.25
	EIR/night	0.06	0.13	3.14	1.80	0.25	0
	EIR/month	1.88	3.75	94.13	54.00	7.50	0
Tanguiéta	IS	0	0.22	0	0	0.06	0
	HBR/night	0.06	1.13	0.38	0.25	1.00	0.13
	EIR/night	0	0.25	0	0.00	0.06	0
	EIR/month	0	7.50	0	0	1.88	0
Natitingou	IS	0.27	0.23	0.08	0	0.08	0
	HBR/night	1.00	0.13	1.00	0.13	1.56	0.13
	EIR/night	0.27	0.03	0.08	0	0.13	0
	EIR/month	8.18	0.87	2.50	0	3.75	0
Kouandé	IS	0.25	0.29	0	0.09	0.11	0
	HBR/night	0.25	1.06	0.88	0.69	0.56	0.25
	EIR/night	0.06	0.31	0	0.06	0.06	0
	EIR/month	1.88	9.38	0	1.88	1.88	0

### Impact of IRS on blood feeding of *Anopheles gambiae* in Atacora

**Fig. 4:**
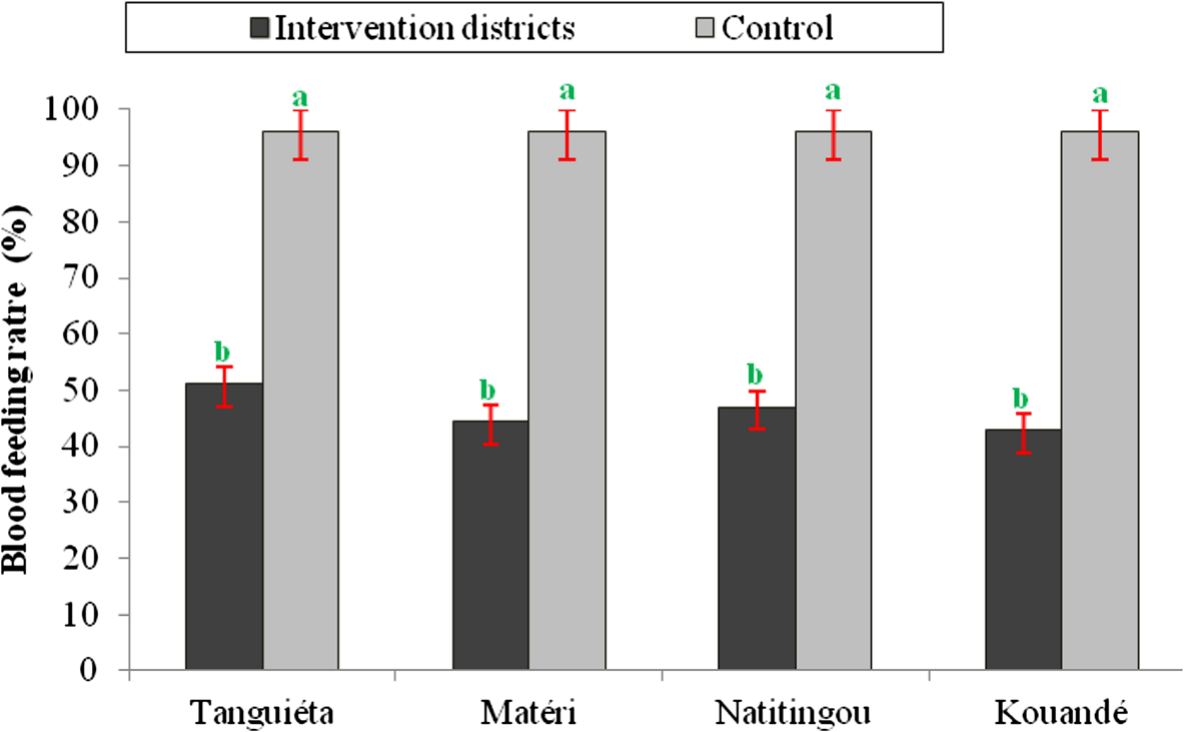
Percentage of blood feeding *Anopheles gambiae* collected in IRS by pyrethrum spray catch (PSC) and in exit windows traps in intervention area and the control. (^a^Value in the same district with the same superscript do not differ significantly by Fisher’s exact test (*p* >0.05)

*Anopheles gambiae* blood-feeding rate observed after each IRS campaign in the treated areas was low (Fig. 4). In 2011, the decrease in blood-feeding rate was significant in treated areas compared to control areas (p <0.05) in all towns (Fig. 4). This rate was 96.05 % in the control town against 42.85 % in Kouandé and 44.3 % in Matéri. It is important to note that a considerable proportion of the few mosquitoes in IRS areas succeeded in feeding on humans. However, the blood-feeding index was significantly lower after IRS.

### Evolution of *Anopheles gambiae* resistance to bendiocarb in Atacora

A monitoring has done on the sensitivity of *An. gambiae* to bendiocarb from 2010 to 2012. After three years, a loss of *An. gambiae* sensitivity to bendiocarb was observed (Table 6). From 2010 to 2012, the mortality rate decreased from 95 % to 78.89 % at Kouande, 97.87 % to 60.27 % at Materi, 96.74 % to 61.90 % at Natitingou, 95.18 % to 79.21 % at Pehunco, 96.84 % to 63.21 % at Tanguiéta and mortality due to bendiocarb was less than 80 % in 2012 in all districts.

**Table 6:** Bendiocarb mortality rate in *Anopheles gambiae* populations from 2010 to 2012 (Atacora)

Districts	Years	n tested	Mortality rate (%)	OR	CI-95 % (OR)	*p*
Kouandé	2010	123	95.00^a^	1.00	-	-
	2011	82	89.00^ab^	0.42	(0.13–1.23)	0.1097
	2012	90	78.89^b^	0.20	(0.07–0.49)	0.0004
Matéri	2010	94	97.87^a^	1.00	-	-
	2011	85	87.06^b^	0.16	(0.02–0.61)	0.0075
	2012	73	60.27^b^	0.04	(0.01–0.12)	<0.0001
Natitingou	2010	92	96.74^a^	1.00	-	-
	2011	89	85.39^b^	0.21	(0.04–0.68)	0.0083
	2012	84	61.90^b^	0.06	(0.01–0.17)	<0.0001
Pehunco	2010	83	95.18^a^	1.00	-	-
	2011	80	93.75^b^	0.71	(0.18–3.11)	0.7431
	2012	101	79.21^b^	0.20	(0.05–0.55)	0.00199
Tanguiéta	2010	95	96.84^a^	1.00	-	-
	2011	82	64.63^b^	0.06	(0.01–0.18)	<0.0001
	2012	106	63.21^b^	0.06	(0.01–0.17)	<0.0001

For each district, numbers in the same column with the same superscript do not differ significantly by Fisher’s exact test (*p* >0.05)

*OR* Odd ratio, *CI* confidence interval, *p-value* probability value

## Discussion

The data presented in this article are a summary of published works [16, 17, 21, 22, 26] from CREC, which is in charge of IRS M&E in Benin. Discussion in this article mainly focusses on the shortcomings of previous work that remained silent about concerns raised by Benin NMCP and the partner funding IRS in Benin. Four main points from the IRS campaigns in Benin deserve special attention: i) the great adhesion from the population; ii) the skill and expertise of the international NGOs responsible for implementation; iii) the high coverage of protected households exceeding 80 % of the sleeping quarters; and, iv) a significant reduction in malaria transmission. These successes have been described in many reports [16, 17, 21, 22, 26]. However, this paper does not emphasize these results but presents the challenges and attempts to suggest solutions.

### Challenge 1**-**reducing IRS cost

This study has not estimated IRS cost in Benin, but when considering the costs reported by the financial partners, it appears relatively high. Very few countries in sub-Saharan African countries are able to afford IRS on their national budgets. Unfortunately, IRS in Africa cannot remain at the expense of international partners forever. If for some reason, the financial partner withdraws, this could rebound in transmission. This is the case for IRS results in the department of Ouémé in Benin: when IRS was moved from Ouémé to Atacora, a resurgence of all malaria entomological indicators was noted a year later [27]. IRS must be made cheaper and more affordable. There are irreducible sections included in IRS cost planning. This is the case when environmental compliance, logistics and insecticide provisioning are involved. However, cost related to staff, management and technical assistance must be reduced. For this reason, the presence of international NGOs supporting the implementation of IRS should be limited in time to allow a local organization with the relevant skills in terms of IRS planning and implementation to take over. Such an organization must ensure a better partnership with the NMCP and the financial partner.

### Challenge 2-Locations appropriate for IRS implementation

The M&E of IRS in Ouémé and Atacora has shown that the decrease in the level of malaria transmission lasted only four months after IRS implementation. Beyond four months, bendiocarb was no longer effective on treated walls. The same applied for pirimiphos methyl EC. In fact, the persistence on treated walls of the two formulations used, Ficam M and Actellic EC, is low. However, in Atacora, malaria transmission is year round. In such a situation, the only alternative while awaiting the development of new products is selecting seasonal transmission zones, i.e., areas where malaria transmission period coincides with the duration of residual effect of the selected insecticide. On the other hand, malaria pre-elimination areas are needed to accelerate elimination. The same alternative applies for periods of high prevalence of malaria or epidemics of malaria in order to reduce the transmission rate.

### Challenge 3-management of *Anopheles* resistance to insecticides

After two years of bendiocarb use in Atacora, the emergence of *An. gambiae* resistance to this product was reported [17, 23, 24]. In this study area, the appearance of this resistance might have been linked not only to the IRS campaign, but also to the massive use of agricultural insecticides against cotton pests [28]. To address bendiocarb resistance, the NMCP selected pirimiphos methyl, another insecticide belonging to the class of organophosphates, to be used for IRS in Atacora. The management of resistance cannot be perceived by replacing one insecticide with another. Two methods are proposed as far as Atacora is concerned: i) in order to weaken the mosquitoes carrying resistance genes (fitness cost), avoid exposing *Anopheles* to the same product over several years. To achieve this, alternation of IRS campaigns with LLIN distribution campaigns. IRS is suggested would then be implemented every three years with two years’ extensive use of LLINs inserted. This means that the implementation of IRS will take place two years after the distribution of LLINs. By that time, most LLINs will have lost their insecticidal effectiveness and some will have been torn. The loss of effectiveness of pyrethroids will then be covered by IRS, based on a carbamate or an organophosphate; and, ii) to be used simultaneously with the first method, a combination of two insecticides with different modes of action to be used in rotation or mosaic system. For example, the intervention zone would be divided into sectors. Each sector would be treated with one of the two products so as to have two adjacent sectors treated with two different products of two different actions. In addition, the insecticide rotation system should be effectively applied year after year.

### Challenge 4-partial IRS + LLIN combination

During the Ouémé and Atacora campaigns, inhabitants in the concerned areas believed that IRS alone was sufficient to ensure their protection against mosquito bites and they did not bother sleeping under mosquito nets. But, when a mosquito succeeds in entering a house, it goes directly to its host and gets its blood meal even when the house is treated. After the meal, the mosquito either remains on the wall or seeks to escape when the house is treated. This situation was explained since fed mosquitoes were collected by pyrethrum spray catch and in exit window traps in treated houses and experimental huts in Malanville [29] as well as at community level [11, 30]. This is the reason why it has been suggested that the NMCP educate communities of Benin protected by IRS, particularly children and pregnant women, to consider sleeping under LLINs to supplement malaria control efforts.

## Conclusion

The choice of IRS as malaria prevention strategy is in line with the MDGs, particularly, the reduction of the morbidity and the mortality related to malaria by 75 % from 2000 to 2015. Reducing the prevalence of malaria by 75 % in 15 years and progressing towards malaria elimination is not an easy task. To achieve malaria elimination, any available means of controlling the disease must be exploited including the control of both vector and parasite. For that purpose, no control strategy should be excluded. IRS must therefore continue in Benin, in areas where the intervention is favourable.
